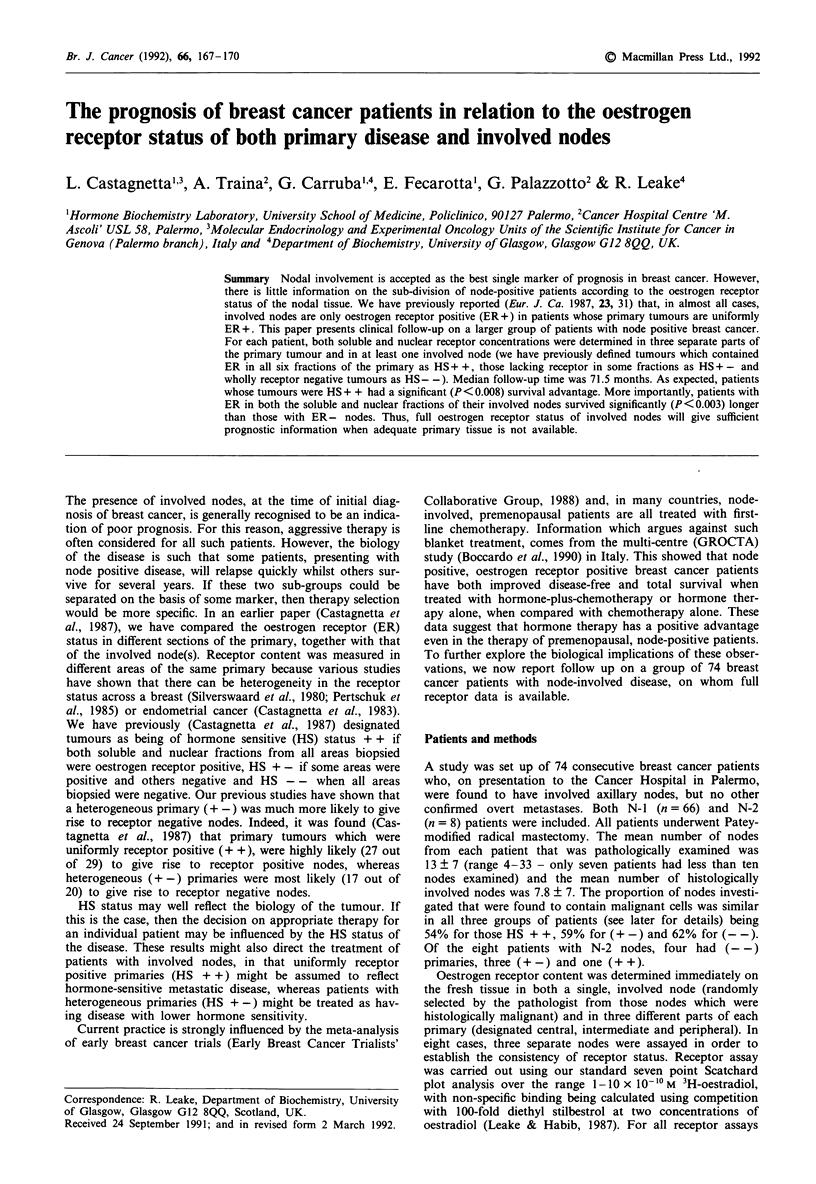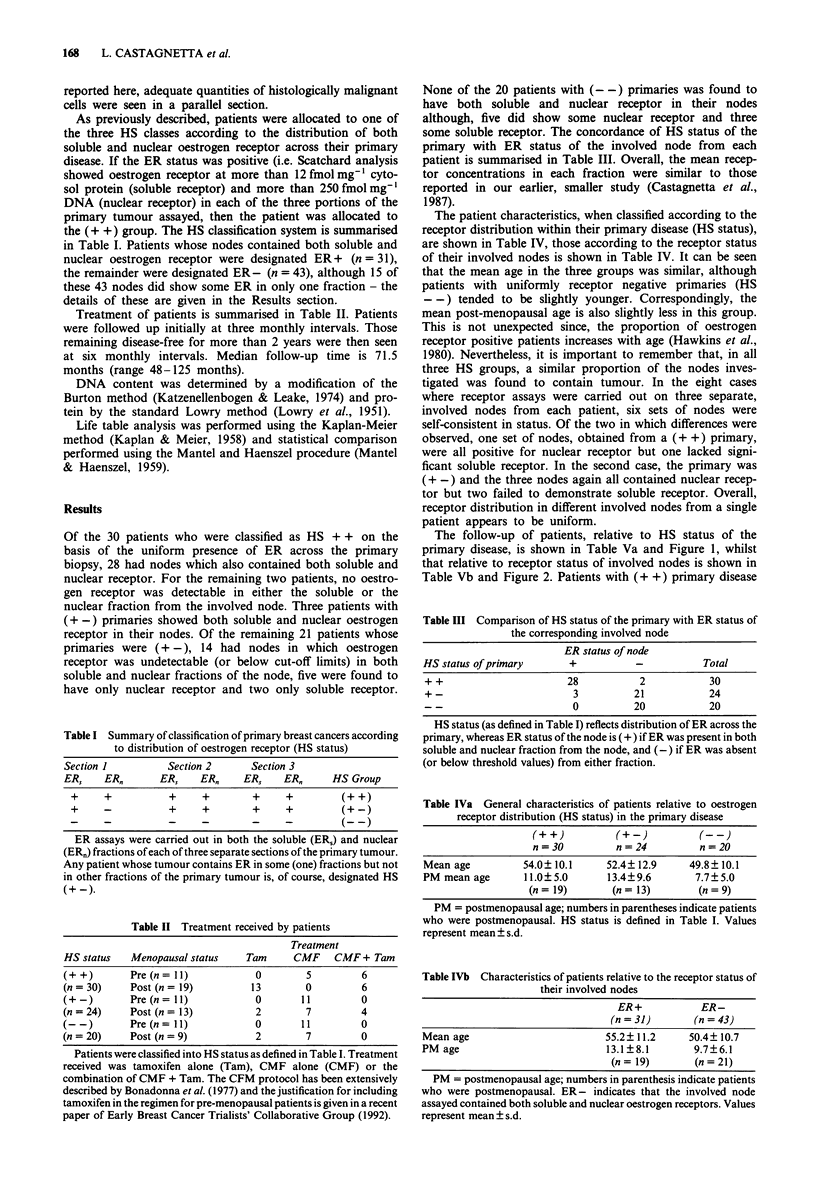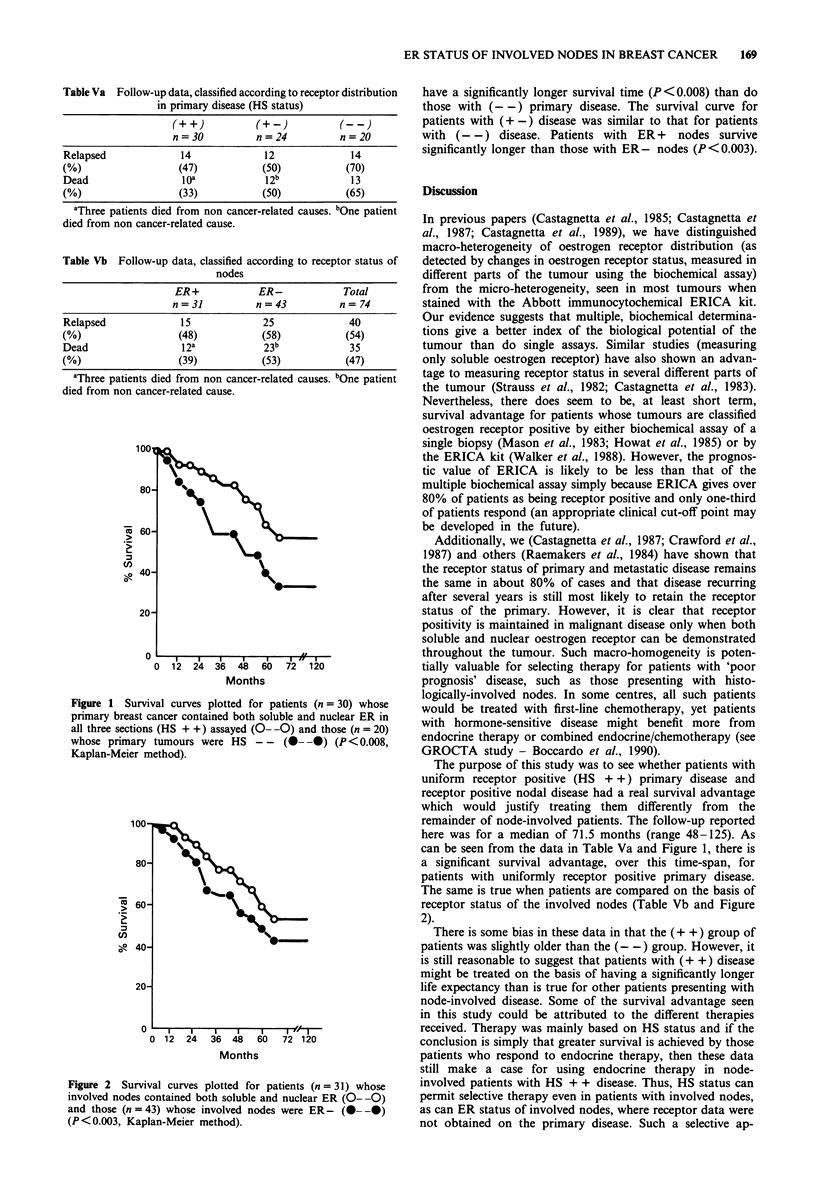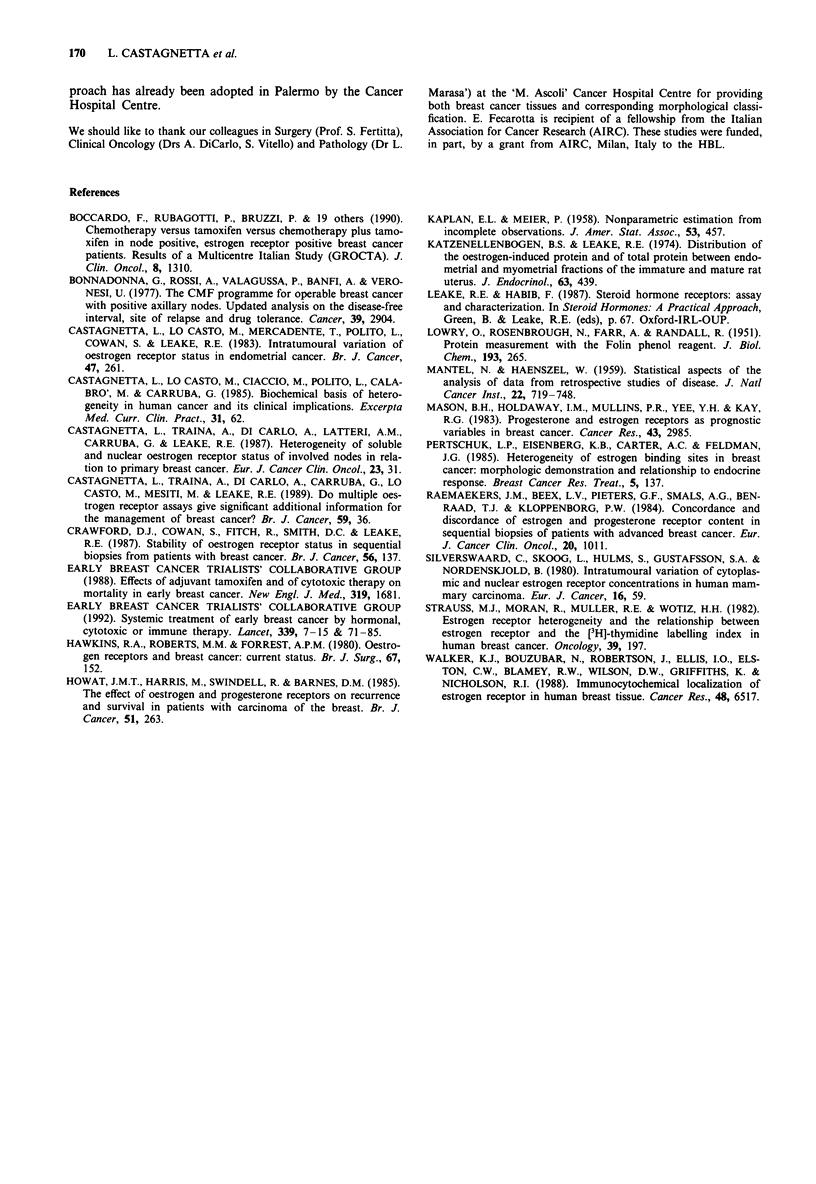# The prognosis of breast cancer patients in relation to the oestrogen receptor status of both primary disease and involved nodes.

**DOI:** 10.1038/bjc.1992.236

**Published:** 1992-07

**Authors:** L. Castagnetta, A. Traina, G. Carruba, E. Fecarotta, G. Palazzotto, R. Leake

**Affiliations:** Hormone Biochemistry Laboratory, University School of Medicine, Policlinico, Palermo, Italy.

## Abstract

Nodal involvement is accepted as the best single marker of prognosis in breast cancer. However, there is little information on the sub-division of node-positive patients according to the oestrogen receptor status of the nodal tissue. We have previously reported (Eur. J. Ca. 1987, 23, 31) that, in almost all cases, involved nodes are only oestrogen receptor positive (ER+) in patients whose primary tumours are uniformly ER+. This paper presents clinical follow-up on a larger group of patients with node positive breast cancer. For each patient, both soluble and nuclear receptor concentrations were determined in three separate parts of the primary tumour and in at least one involved node (we have previously defined tumours which contained ER in all six fractions of the primary as HS++, those lacking receptor in some fractions as HS+- and wholly receptor negative tumours as HS--). Median follow-up time was 71.5 months. As expected, patients whose tumours were HS++ had a significant (P less than 0.008) survival advantage. More importantly, patients with ER in both the soluble and nuclear fractions of their involved nodes survived significantly (P less than 0.003) longer than those with ER- nodes. Thus, full oestrogen receptor status of involved nodes will give sufficient prognostic information when adequate primary tissue is not available.


*Br. J. Cancer* (1992), **66**, 167–170 © Macmillan Press Ltd., 1992

### The prognosis of breast cancer patients in relation to the oestrogen receptor status of both primary disease and involved nodes

L. Castagnetta[1,3], A. Traina[2], G. Carruba[1,4], E. Fecarotta[1], G. Palazzotto[2] & R. Leake[4]

[1]*Hormone Biochemistry Laboratory, University School of Medicine, Policlinico, 90127 Palermo,* [2]*Cancer Hospital Centre 'M. Ascoli' USL 58, Palermo,* [3]*Molecular Endocrinology and Experimental Oncology Units of the Scientific Institute for Cancer in Genova (Palermo branch), Italy and* [4]*Department of Biochemistry, University of Glasgow, Glasgow G12 8QQ, UK.*

**Summary** Nodal involvement is accepted as the best single marker of prognosis in breast cancer. However, there is little information on the sub-division of node-positive patients according to the oestrogen receptor status of the nodal tissue. We have previously reported (*Eur. J. Ca.* 1987, **23**, 31) that, in almost all cases, involved nodes are only oestrogen receptor positive (ER+) in patients whose primary tumours are uniformly ER+. This paper presents clinical follow-up on a larger group of patients with node positive breast cancer. For each patient, both soluble and nuclear receptor concentrations were determined in three separate parts of the primary tumour and in at least one involved node (we have previously defined tumours which contained ER in all six fractions of the primary as HS++, those lacking receptor in some fractions as HS+− and wholly receptor negative tumours as HS−−). Median follow-up time was 71.5 months. As expected, patients whose tumours were HS++ had a significant ($P<0.008$) survival advantage. More importantly, patients with ER in both the soluble and nuclear fractions of their involved nodes survived significantly ($P<0.003$) longer than those with ER− nodes. Thus, full oestrogen receptor status of involved nodes will give sufficient prognostic information when adequate primary tissue is not available.

The presence of involved nodes, at the time of initial diagnosis of breast cancer, is generally recognised to be an indication of poor prognosis. For this reason, aggressive therapy is often considered for all such patients. However, the biology of the disease is such that some patients, presenting with node positive disease, will relapse quickly whilst others survive for several years. If these two sub-groups could be separated on the basis of some marker, then therapy selection would be more specific. In an earlier paper (Castagnetta *et al*., 1987), we have compared the oestrogen receptor (ER) status in different sections of the primary, together with that of the involved node(s). Receptor content was measured in different areas of the same primary because various studies have shown that there can be heterogeneity in the receptor status across a breast (Silverswaard *et al*., 1980; Pertschuk *et al*., 1985) or endometrial cancer (Castagnetta *et al*., 1983). We have previously (Castagnetta *et al*., 1987) designated tumours as being of hormone sensitive (HS) status ++ if both soluble and nuclear fractions from all areas biopsied were oestrogen receptor positive, HS +− if some areas were positive and others negative and HS −− when all areas biopsied were negative. Our previous studies have shown that a heterogeneous primary (+−) was much more likely to give rise to receptor negative nodes. Indeed, it was found (Castagnetta *et al*., 1987) that primary tumours which were uniformly receptor positive (++), were highly likely (27 out of 29) to give rise to receptor positive nodes, whereas heterogeneous (+−) primaries were most likely (17 out of 20) to give rise to receptor negative nodes.

HS status may well reflect the biology of the tumour. If this is the case, then the decision on appropriate therapy for an individual patient may be influenced by the HS status of the disease. These results might also direct the treatment of patients with involved nodes, in that uniformly receptor positive primaries (HS ++) might be assumed to reflect hormone-sensitive metastatic disease, whereas patients with heterogeneous primaries (HS +−) might be treated as having disease with lower hormone sensitivity.

Current practice is strongly influenced by the meta-analysis of early breast cancer trials (Early Breast Cancer Trialists' Collaborative Group, 1988) and, in many countries, node-involved, premenopausal patients are all treated with first-line chemotherapy. Information which argues against such blanket treatment, comes from the multi-centre (GROCTA) study (Boccardo *et al*., 1990) in Italy. This showed that node positive, oestrogen receptor positive breast cancer patients have both improved disease-free and total survival when treated with hormone-plus-chemotherapy or hormone therapy alone, when compared with chemotherapy alone. These data suggest that hormone therapy has a positive advantage even in the therapy of premenopausal, node-positive patients. To further explore the biological implications of these observations, we now report follow up on a group of 74 breast cancer patients with node-involved disease, on whom full receptor data is available.

### Patients and methods

A study was set up of 74 consecutive breast cancer patients who, on presentation to the Cancer Hospital in Palermo, were found to have involved axillary nodes, but no other confirmed overt metastases. Both N-1 ($n = 66$) and N-2 ($n = 8$) patients were included. All patients underwent Patey-modified radical mastectomy. The mean number of nodes from each patient that was pathologically examined was $13 \pm 7$ (range 4–33 – only seven patients had less than ten nodes examined) and the mean number of histologically involved nodes was $7.8 \pm 7$. The proportion of nodes investigated that were found to contain malignant cells was similar in all three groups of patients (see later for details) being 54% for those HS ++, 59% for (+−) and 62% for (−−). Of the eight patients with N-2 nodes, four had (−−) primaries, three (+−) and one (++).

Oestrogen receptor content was determined immediately on the fresh tissue in both a single, involved node (randomly selected by the pathologist from those nodes which were histologically malignant) and in three different parts of each primary (designated central, intermediate and peripheral). In eight cases, three separate nodes were assayed in order to establish the consistency of receptor status. Receptor assay was carried out using our standard seven point Scatchard plot analysis over the range $1-10 \times 10^{-10}$ M $^3$H-oestradiol, with non-specific binding being calculated using competition with 100-fold diethyl stilbestrol at two concentrations of oestradiol (Leake & Habib, 1987). For all receptor assays

Correspondence: R. Leake, Department of Biochemistry, University of Glasgow, Glasgow G12 8QQ, Scotland, UK.
Received 24 September 1991; and in revised form 2 March 1992.

reported here, adequate quantities of histologically malignant cells were seen in a parallel section.

As previously described, patients were allocated to one of the three HS classes according to the distribution of both soluble and nuclear oestrogen receptor across their primary disease. If the ER status was positive (i.e. Scatchard analysis showed oestrogen receptor at more than 12 fmol $mg^{-1}$ cytosol protein (soluble receptor) and more than 250 fmol $mg^{-1}$ DNA (nuclear receptor) in each of the three portions of the primary tumour assayed, then the patient was allocated to the (+ +) group. The HS classification system is summarised in Table I. Patients whose nodes contained both soluble and nuclear oestrogen receptor were designated ER+ ($n = 31$), the remainder were designated ER− ($n = 43$), although 15 of these 43 nodes did show some ER in only one fraction – the details of these are given in the Results section.

Treatment of patients is summarised in Table II. Patients were followed up initially at three monthly intervals. Those remaining disease-free for more than 2 years were then seen at six monthly intervals. Median follow-up time is 71.5 months (range 48–125 months).

DNA content was determined by a modification of the Burton method (Katzenellenbogen & Leake, 1974) and protein by the standard Lowry method (Lowry et al., 1951).

Life table analysis was performed using the Kaplan-Meier method (Kaplan & Meier, 1958) and statistical comparison performed using the Mantel and Haenszel procedure (Mantel & Haenszel, 1959).

### Results

Of the 30 patients who were classified as HS + + on the basis of the uniform presence of ER across the primary biopsy, 28 had nodes which also contained both soluble and nuclear receptor. For the remaining two patients, no oestrogen receptor was detectable in either the soluble or the nuclear fraction from the involved node. Three patients with (+ −) primaries showed both soluble and nuclear oestrogen receptor in their nodes. Of the remaining 21 patients whose primaries were (+ −), 14 had nodes in which oestrogen receptor was undetectable (or below cut-off limits) in both soluble and nuclear fractions of the node, five were found to have only nuclear receptor and two only soluble receptor.

**Table I** Summary of classification of primary breast cancers according to distribution of oestrogen receptor (HS status)

| Section 1 | | Section 2 | | Section 3 | | |
|---|---|---|---|---|---|---|
| $ER_s$ | $ER_n$ | $ER_s$ | $ER_n$ | $ER_s$ | $ER_n$ | HS Group |
| + | + | + | + | + | + | (+ +) |
| + | − | + | + | + | + | (+ −) |
| − | − | − | − | − | − | (− −) |

ER assays were carried out in both the soluble ($ER_s$) and nuclear ($ER_n$) fractions of each of three separate sections of the primary tumour. Any patient whose tumour contains ER in some (one) fractions but not in other fractions of the primary tumour is, of course, designated HS (+ −).

**Table II** Treatment received by patients

| HS status | Menopausal status | Tam | Treatment CMF | CMF + Tam |
|---|---|---|---|---|
| (+ +) | Pre ($n$ = 11) | 0 | 5 | 6 |
| ($n$ = 30) | Post ($n$ = 19) | 13 | 0 | 6 |
| (+ −) | Pre ($n$ = 11) | 0 | 11 | 0 |
| ($n$ = 24) | Post ($n$ = 13) | 2 | 7 | 4 |
| (− −) | Pre ($n$ = 11) | 0 | 11 | 0 |
| ($n$ = 20) | Post ($n$ = 9) | 2 | 7 | 0 |

Patients were classified into HS status as defined in Table I. Treatment received was tamoxifen alone (Tam), CMF alone (CMF) or the combination of CMF + Tam. The CFM protocol has been extensively described by Bonadonna et al. (1977) and the justification for including tamoxifen in the regimen for pre-menopausal patients is given in a recent paper of Early Breast Cancer Trialists' Collaborative Group (1992).

None of the 20 patients with (− −) primaries was found to have both soluble and nuclear receptor in their nodes although, five did show some nuclear receptor and three some soluble receptor. The concordance of HS status of the primary with ER status of the involved node from each patient is summarised in Table III. Overall, the mean receptor concentrations in each fraction were similar to those reported in our earlier, smaller study (Castagnetta et al., 1987).

The patient characteristics, when classified according to the receptor distribution within their primary disease (HS status), are shown in Table IV, those according to the receptor status of their involved nodes is shown in Table IV. It can be seen that the mean age in the three groups was similar, although patients with uniformly receptor negative primaries (HS − −) tended to be slightly younger. Correspondingly, the mean post-menopausal age is also slightly less in this group. This is not unexpected since, the proportion of oestrogen receptor positive patients increases with age (Hawkins et al., 1980). Nevertheless, it is important to remember that, in all three HS groups, a similar proportion of the nodes investigated was found to contain tumour. In the eight cases where receptor assays were carried out on three separate, involved nodes from each patient, six sets of nodes were self-consistent in status. Of the two in which differences were observed, one set of nodes, obtained from a (+ +) primary, were all positive for nuclear receptor but one lacked significant soluble receptor. In the second case, the primary was (+ −) and the three nodes again all contained nuclear receptor but two failed to demonstrate soluble receptor. Overall, receptor distribution in different involved nodes from a single patient appears to be uniform.

The follow-up of patients, relative to HS status of the primary disease, is shown in Table Va and Figure 1, whilst that relative to receptor status of involved nodes is shown in Table Vb and Figure 2. Patients with (+ +) primary disease

**Table III** Comparison of HS status of the primary with ER status of the corresponding involved node

| HS status of primary | ER status of node + | − | Total |
|---|---|---|---|
| + + | 28 | 2 | 30 |
| + − | 3 | 21 | 24 |
| − − | 0 | 20 | 20 |

HS status (as defined in Table I) reflects distribution of ER across the primary, whereas ER status of the node is (+) if ER was present in both soluble and nuclear fraction from the node, and (−) if ER was absent (or below threshold values) from either fraction.

**Table IVa** General characteristics of patients relative to oestrogen receptor distribution (HS status) in the primary disease

| | (+ +) $n$ = 30 | (+ −) $n$ = 24 | (− −) $n$ = 20 |
|---|---|---|---|
| Mean age | 54.0 ± 10.1 | 52.4 ± 12.9 | 49.8 ± 10.1 |
| PM mean age | 11.0 ± 5.0 | 13.4 ± 9.6 | 7.7 ± 5.0 |
| | ($n$ = 19) | ($n$ = 13) | ($n$ = 9) |

PM = postmenopausal age; numbers in parentheses indicate patients who were postmenopausal. HS status is defined in Table I. Values represent mean ± s.d.

**Table IVb** Characteristics of patients relative to the receptor status of their involved nodes

| | ER+ ($n$ = 31) | ER− ($n$ = 43) |
|---|---|---|
| Mean age | 55.2 ± 11.2 | 50.4 ± 10.7 |
| PM age | 13.1 ± 8.1 | 9.7 ± 6.1 |
| | ($n$ = 19) | ($n$ = 21) |

PM = postmenopausal age; numbers in parenthesis indicate patients who were postmenopausal. ER− indicates that the involved node assayed contained both soluble and nuclear oestrogen receptors. Values represent mean ± s.d.

**Table Va** Follow-up data, classified according to receptor distribution in primary disease (HS status)

| | (++) n = 30 | (+−) n = 24 | (−−) n = 20 |
|---|---|---|---|
| Relapsed | 14 | 12 | 14 |
| (%) | (47) | (50) | (70) |
| Dead | 10[a] | 12[b] | 13 |
| (%) | (33) | (50) | (65) |

[a]Three patients died from non cancer-related causes. [b]One patient died from non cancer-related cause.

**Table Vb** Follow-up data, classified according to receptor status of nodes

| | ER+ n = 31 | ER− n = 43 | Total n = 74 |
|---|---|---|---|
| Relapsed | 15 | 25 | 40 |
| (%) | (48) | (58) | (54) |
| Dead | 12[a] | 23[b] | 35 |
| (%) | (39) | (53) | (47) |

[a]Three patients died from non cancer-related causes. [b]One patient died from non cancer-related cause.

**Figure 1** Survival curves plotted for patients ($n = 30$) whose primary breast cancer contained both soluble and nuclear ER in all three sections (HS + +) assayed (O--O) and those ($n = 20$) whose primary tumours were HS − − (●--●) ($P < 0.008$, Kaplan-Meier method).

**Figure 2** Survival curves plotted for patients ($n = 31$) whose involved nodes contained both soluble and nuclear ER (O--O) and those ($n = 43$) whose involved nodes were ER− (●--●) ($P < 0.003$, Kaplan-Meier method).

have a significantly longer survival time ($P < 0.008$) than do those with (− −) primary disease. The survival curve for patients with (+ −) disease was similar to that for patients with (− −) disease. Patients with ER+ nodes survive significantly longer than those with ER− nodes ($P < 0.003$).

### Discussion

In previous papers (Castagnetta *et al.*, 1985; Castagnetta *et al.*, 1987; Castagnetta *et al.*, 1989), we have distinguished macro-heterogeneity of oestrogen receptor distribution (as detected by changes in oestrogen receptor status, measured in different parts of the tumour using the biochemical assay) from the micro-heterogeneity, seen in most tumours when stained with the Abbott immunocytochemical ERICA kit. Our evidence suggests that multiple, biochemical determinations give a better index of the biological potential of the tumour than do single assays. Similar studies (measuring only soluble oestrogen receptor) have also shown an advantage to measuring receptor status in several different parts of the tumour (Strauss *et al.*, 1982; Castagnetta *et al.*, 1983). Nevertheless, there does seem to be, at least short term, survival advantage for patients whose tumours are classified oestrogen receptor positive by either biochemical assay of a single biopsy (Mason *et al.*, 1983; Howat *et al.*, 1985) or by the ERICA kit (Walker *et al.*, 1988). However, the prognostic value of ERICA is likely to be less than that of the multiple biochemical assay simply because ERICA gives over 80% of patients as being receptor positive and only one-third of patients respond (an appropriate clinical cut-off point may be developed in the future).

Additionally, we (Castagnetta *et al.*, 1987; Crawford *et al.*, 1987) and others (Raemakers *et al.*, 1984) have shown that the receptor status of primary and metastatic disease remains the same in about 80% of cases and that disease recurring after several years is still most likely to retain the receptor status of the primary. However, it is clear that receptor positivity is maintained in malignant disease only when both soluble and nuclear oestrogen receptor can be demonstrated throughout the tumour. Such macro-homogeneity is potentially valuable for selecting therapy for patients with 'poor prognosis' disease, such as those presenting with histologically-involved nodes. In some centres, all such patients would be treated with first-line chemotherapy, yet patients with hormone-sensitive disease might benefit more from endocrine therapy or combined endocrine/chemotherapy (see GROCTA study – Boccardo *et al.*, 1990).

The purpose of this study was to see whether patients with uniform receptor positive (HS + +) primary disease and receptor positive nodal disease had a real survival advantage which would justify treating them differently from the remainder of node-involved patients. The follow-up reported here was for a median of 71.5 months (range 48–125). As can be seen from the data in Table Va and Figure 1, there is a significant survival advantage, over this time-span, for patients with uniformly receptor positive primary disease. The same is true when patients are compared on the basis of receptor status of the involved nodes (Table Vb and Figure 2).

There is some bias in these data in that the (+ +) group of patients was slightly older than the (− −) group. However, it is still reasonable to suggest that patients with (+ +) disease might be treated on the basis of having a significantly longer life expectancy than is true for other patients presenting with node-involved disease. Some of the survival advantage seen in this study could be attributed to the different therapies received. Therapy was mainly based on HS status and if the conclusion is simply that greater survival is achieved by those patients who respond to endocrine therapy, then these data still make a case for using endocrine therapy in node-involved patients with HS + + disease. Thus, HS status can permit selective therapy even in patients with involved nodes, as can ER status of involved nodes, where receptor data were not obtained on the primary disease. Such a selective approach has already been adopted in Palermo by the Cancer Hospital Centre.

We should like to thank our colleagues in Surgery (Prof. S. Fertitta), Clinical Oncology (Drs A. DiCarlo, S. Vitello) and Pathology (Dr L. Marasa') at the 'M. Ascoli' Cancer Hospital Centre for providing both breast cancer tissues and corresponding morphological classification. E. Fecarotta is recipient of a fellowship from the Italian Association for Cancer Research (AIRC). These studies were funded, in part, by a grant from AIRC, Milan, Italy to the HBL.

### References

BOCCARDO, F., RUBAGOTTI, P., BRUZZI, P. & 19 others (1990). Chemotherapy versus tamoxifen versus chemotherapy plus tamoxifen in node positive, estrogen receptor positive breast cancer patients. Results of a Multicentre Italian Study (GROCTA). *J. Clin. Oncol.*, **8**, 1310.

BONNADONNA, G., ROSSI, A., VALAGUSSA, P., BANFI, A. & VERONESI, U. (1977). The CMF programme for operable breast cancer with positive axillary nodes. Updated analysis on the disease-free interval, site of relapse and drug tolerance. *Cancer*, **39**, 2904.

CASTAGNETTA, L., LO CASTO, M., MERCADENTE, T., POLITO, L., COWAN, S. & LEAKE, R.E. (1983). Intratumoural variation of oestrogen receptor status in endometrial cancer. *Br. J. Cancer*, **47**, 261.

CASTAGNETTA, L., LO CASTO, M., CIACCIO, M., POLITO, L., CALABRO', M. & CARRUBA, G. (1985). Biochemical basis of heterogeneity in human cancer and its clinical implications. *Excerpta Med. Curr. Clin. Pract.*, **31**, 62.

CASTAGNETTA, L., TRAINA, A., DI CARLO, A., LATTERI, A.M., CARRUBA, G. & LEAKE, R.E. (1987). Heterogeneity of soluble and nuclear oestrogen receptor status of involved nodes in relation to primary breast cancer. *Eur. J. Cancer Clin. Oncol.*, **23**, 31.

CASTAGNETTA, L., TRAINA, A., DI CARLO, A., CARRUBA, G., LO CASTO, M., MESITI, M. & LEAKE, R.E. (1989). Do multiple oestrogen receptor assays give significant additional information for the management of breast cancer? *Br. J. Cancer*, **59**, 36.

CRAWFORD, D.J., COWAN, S., FITCH, R., SMITH, D.C. & LEAKE, R.E. (1987). Stability of oestrogen receptor status in sequential biopsies from patients with breast cancer. *Br. J. Cancer*, **56**, 137.

EARLY BREAST CANCER TRIALISTS' COLLABORATIVE GROUP (1988). Effects of adjuvant tamoxifen and of cytotoxic therapy on mortality in early breast cancer. *New Engl. J. Med.*, **319**, 1681.

EARLY BREAST CANCER TRIALISTS' COLLABORATIVE GROUP (1992). Systemic treatment of early breast cancer by hormonal, cytotoxic or immune therapy. *Lancet*, **339**, 7-15 & 71-85.

HAWKINS, R.A., ROBERTS, M.M. & FORREST, A.P.M. (1980). Oestrogen receptors and breast cancer: current status. *Br. J. Surg.*, **67**, 152.

HOWAT, J.M.T., HARRIS, M., SWINDELL, R. & BARNES, D.M. (1985). The effect of oestrogen and progesterone receptors on recurrence and survival in patients with carcinoma of the breast. *Br. J. Cancer*, **51**, 263.

KAPLAN, E.L. & MEIER, P. (1958). Nonparametric estimation from incomplete observations. *J. Amer. Stat. Assoc.*, **53**, 457.

KATZENELLENBOGEN, B.S. & LEAKE, R.E. (1974). Distribution of the oestrogen-induced protein and of total protein between endometrial and myometrial fractions of the immature and mature rat uterus. *J. Endocrinol.*, **63**, 439.

LEAKE, R.E. & HABIB, F. (1987). Steroid hormone receptors: assay and characterization. In *Steroid Hormones: A Practical Approach*, Green, B. & Leake, R.E. (eds), p. 67. Oxford-IRL-OUP.

LOWRY, O., ROSENBROUGH, N., FARR, A. & RANDALL, R. (1951). Protein measurement with the Folin phenol reagent. *J. Biol. Chem.*, **193**, 265.

MANTEL, N. & HAENSZEL, W. (1959). Statistical aspects of the analysis of data from retrospective studies of disease. *J. Natl Cancer Inst.*, **22**, 719-748.

MASON, B.H., HOLDAWAY, I.M., MULLINS, P.R., YEE, Y.H. & KAY, R.G. (1983). Progesterone and estrogen receptors as prognostic variables in breast cancer. *Cancer Res.*, **43**, 2985.

PERTSCHUK, L.P., EISENBERG, K.B., CARTER, A.C. & FELDMAN, J.G. (1985). Heterogeneity of estrogen binding sites in breast cancer: morphologic demonstration and relationship to endocrine response. *Breast Cancer Res. Treat.*, **5**, 137.

RAEMAEKERS, J.M., BEEX, L.V., PIETERS, G.F., SMALS, A.G., BENRAAD, T.J. & KLOPPENBORG, P.W. (1984). Concordance and discordance of estrogen and progesterone receptor content in sequential biopsies of patients with advanced breast cancer. *Eur. J. Cancer Clin. Oncol.*, **20**, 1011.

SILVERSWAARD, C., SKOOG, L., HULMS, S., GUSTAFSSON, S.A. & NORDENSKJOLD, B. (1980). Intratumoural variation of cytoplasmic and nuclear estrogen receptor concentrations in human mammary carcinoma. *Eur. J. Cancer*, **16**, 59.

STRAUSS, M.J., MORAN, R., MULLER, R.E. & WOTIZ, H.H. (1982). Estrogen receptor heterogeneity and the relationship between estrogen receptor and the [$^3$H]-thymidine labelling index in human breast cancer. *Oncology*, **39**, 197.

WALKER, K.J., BOUZUBAR, N., ROBERTSON, J., ELLIS, I.O., ELSTON, C.W., BLAMEY, R.W., WILSON, D.W., GRIFFITHS, K. & NICHOLSON, R.I. (1988). Immunocytochemical localization of estrogen receptor in human breast tissue. *Cancer Res.*, **48**, 6517.